# Physical Activity and BrainAGE: Exploring the Impact on Brain Health and Plasticity in Older Adults

**DOI:** 10.1002/hbm.70378

**Published:** 2025-10-14

**Authors:** Tannaz Saraei, Simon Schrenk, Christian Puta, Marco Herbsleb, Otto W. Witte, Christiane Frahm, Stefan Brodoehl, Kathrin Finke, Christian Gaser

**Affiliations:** ^1^ Department of Neurology Jena University Hospital – Friedrich Schiller University of Jena Jena Germany; ^2^ Department of Sports Medicine and Health Promotion Friedrich‐Schiller‐University Jena Jena Germany; ^3^ Center for Interdisciplinary Prevention of Diseases Related to Professional Activities Friedrich‐Schiller‐University Jena Jena Germany; ^4^ Center for Sepsis Control and Care (CSCC) Jena University Hospital – Friedrich Schiller University of Jena Jena Germany; ^5^ Department of Psychosomatic Medicine Jena University Hospital – Friedrich Schiller University of Jena Jena Germany; ^6^ Department of Psychiatry and Psychotherapy Jena University Hospital – Friedrich Schiller University of Jena Jena Germany; ^7^ German Center for Mental Health (DZPG) Jena Germany

**Keywords:** brain plasticity, brainAGE, life style, physical activity, structural MRI

## Abstract

With an aging global population, cognitive decline in older adults presents significant healthcare challenges. Emerging evidence suggests that physical activity can support cognitive health by promoting plasticity, functional reorganization, and structural adaptation of the brain. In the FIT4BRAIN study, we examined the effects of multi‐component physical activity on cognitive and brain health. Here, we report the results on one of the secondary outcomes, namely changes in brain age (BrainAGE), which estimates the difference between chronological and predicted brain age based on structural MRI data, and changes in brain structure, assessed through voxel‐based morphometry (VBM). Ninety‐two healthy older adults were randomized into a multi‐component physical activity group, performing aerobic, coordination, and balance exercises, or an active control group engaging in non‐aerobic relaxation exercises and educational content (physical activity group (PAG): 36 participants; active control group (CON): 33 participants). Of these, 69 participants underwent MRI assessment and were included in the present analyses. BrainAGE analyses revealed a greater decrease in the physical activity group compared to the control group, indicating a beneficial effect of physical activity on brain aging. Subgroup analyses based on baseline cardiorespiratory fitness (CRF) further revealed that participants with lower CRF showed greater benefits, consistent with VBM findings of structural changes in the same subgroup. These results underscore BrainAGE as a sensitive biomarker for intervention outcomes and suggest that stratification by baseline fitness level may help identify differences in the benefits of physical activity on brain health.

## Graphical abstract

An eight‐week multi‐component physical activity intervention reduced brain age (BrainAGE) in older adults. The effect was strongest in participants with lower baseline fitness, supported by structural brain changes in motivation‐related regions. Findings support BrainAGE as a sensitive biomarker of intervention effects on brain health.

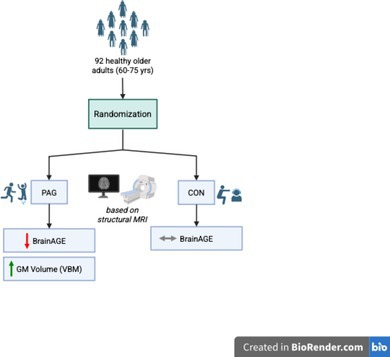

## 1 Introduction

The global increase in the aging population, expected to exceed 1.4 billion by 2030 and 2.1 billion by 2050 (WHO, n.d.; https://www.who.int/news‐room/fact‐sheets/detail/ageing‐and‐health), has raised concerns about age‐related cognitive decline. Cognitive impairments significantly reduce the quality of life in older adults, highlighting the need for interventions that enhance neuroprotection (Deary et al. 2009; International and Patterson 2018). Evidence suggests that neurocognitive disorders emerge from pathological processes that impair the neuronal environment and disrupt brain plasticity. In contrast, beneficial lifestyle factors, particularly physical activity, may support neuroprotection by enhancing brain plasticity (McDonough and Allen 2019; Romanò et al. 2023).

Neuroimaging studies have reported positive associations between physical activity and brain volumes in regions responsible for essential cognitive functions. However, since much of this evidence stems from cross‐sectional studies, findings from randomized controlled trials (RCTs) are still limited. A recent large‐scale analysis across five European cohorts found no significant association between self‐reported physical activity and volumes in key brain regions, raising concerns about the sensitivity of such measures for capturing brain‐activity relationships (Demnitz et al. 2025). This underscores the need for more rigorous experimental designs to establish causality and deepen our understanding of the relationship between CRF and brain health. Our study directly addresses this gap by examining the effects of a structured physical activity intervention on brain structure and aging in older adults using sensitive biomarkers such as BrainAGE (Kalc et al. 2024). BrainAGE estimates the difference between an individual's chronological age and the predicted age of their brain based on structural MRI data, providing a marker of brain health and aging (Kalc et al. 2024).

One potential mechanism linking physical activity to brain health is its impact on cardiorespiratory fitness (CRF). CRF can be measured using maximal oxygen intake (V̇O_2max_), which reflects the maximum rate of oxygen consumption during intense exercise. V̇O_2max_ is a well‐established marker of physical fitness and cardiovascular health (Colcombe et al. 2006). Although higher V̇O_2max_ is associated with better overall health outcomes (Harber et al. 2017), its relationship with brain structure and brain aging remains less well understood.

In older adults, studies have shown a positive association between CRF and cortical gray matter regions including the prefrontal cortex, anterior cingulate, lateral parietal, and lateral temporal cortex (Babaei and Azari 2022; Draganski et al. 2004; Erickson et al. 2014; Gordon et al. 2008; Loprinzi et al. 2021; van Praag et al. 2000; Weinstein et al. 2012). A longitudinal study by Erickson et al. (2010) with healthy older participants found that physical activity was associated with increased brain volume in several regions after a 9‐year follow‐up. Recent findings also suggest that higher physical fitness is linked to better white matter integrity and microstructure (Colcombe et al. 2006; Gordon et al. 2008; Tian et al. 2022).

Recent advances in non‐invasive brain imaging and computational methods have bolstered research on brain health and its structural alterations related to physical activity (Silk and Wood 2011). A promising avenue is using biological age markers like “Brain Age Gap Estimation (BrainAGE),” derived from MRI data in a machine‐learning framework (Colcombe et al. 2006; Franke et al. 2010, 2012, 2014; Franke and Gaser 2012; Franke and Gaser 2019). BrainAGE provides unique insights into individual brain health by estimating BrainAGE and revealing aging patterns (Jylhävä et al. 2017; Ludwig and Smoke 1980). Bittner et al. (Bittner et al. 2021) showed that lifestyle habits significantly impact brain aging, offering insights into why aging varies even among those without neurodegenerative diseases. Sanders et al. (2021) found a positive association between daily steps and a younger‐appearing brain, particularly in women. However, other studies, such as by Wing et al. (2023), found no significant effect of exercise on BrainAGE, highlighting the variability in findings. Nevertheless, despite the potential of these biomarkers, their associations with regular physical activity and brain structure remain unclear.

Here, we report the effects of regular multi‐component physical activity exercise consisting of aerobic, balancing, and coordination exercises, applied in the monocentric, single‐blinded, randomized activity‐controlled “FIT4BRAIN” trial.

Details of the study are given in the published protocol (Schrenk et al. 2023). The trial was registered under https://drks.de/search/de/trial/DRKS00028022. For effects on the primary (cognitive) and functional MRI measures see Schrenk et al. (preprint) (Schrenk et al. 2025). In this paper, we focus on secondary outcomes, specifically brain structure and the aging process, assessed using the BrainAGE biomarker and voxel‐based morphometry (VBM). Given the hypothesized role of baseline cardiorespiratory fitness (V̇O_2max_) in modulating neuroplasticity, we applied both whole‐group and stratified analyses based on baseline V̇O_2max_ to evaluate whether initial fitness levels influenced the brain's response to the intervention. This approach allowed us to test our primary hypothesis while also exploring individual variability in training responsiveness.

## 2 Results

### 2.1 Participant Characteristics

Of the 92 participants initially randomized, 69 underwent MRI assessment and were included in the present analyses (physical activity group (PAG): 36 participants; active control group (CON): 33 participants). Due to missing data, BrainAGE analyses were based on 68 participants, while all other analyses, including V̇O_2_max and brain volume measures, were conducted with 69 participants. As the current analysis was performed on a subset of the original sample, we tested for differences in baseline characteristics between groups. The randomization procedure resulted in comparable groups with respect to age, BMI, years of education, ACE‐III scores, and sex distribution (see Table 1).

**TABLE 1:** Baseline demographic and cognitive characteristics of the physical activity (PAG) and active control (CON) groups (subset with MRI data).

	Physical activity group (*n* = 36)	Active control group (*n* = 33)
Sex (female) (%)	66.6 (24)	66.6 (22)
Age (years)	66.1 ± 3.3	66.4 ± 4
BMI ($\frac{m}{x2}$)	25.8 ± 3.5	26 ± 4
Education (years)	14.9 ± 3.4	14.5 ± 3.5
ACE‐III	94.4 ± 2.8	94.2 ± 2.7

*Note:* Values are presented as mean ± standard deviation unless otherwise noted.

Abbreviations: ACE‐III, Addenbrooke's Cognitive Examination III; PAG, physical activity group; CON, active control group.

### 2.2 Pre and Post‐Intervention Measures

In the whole sample, no significant group differences were found in the change of cardiorespiratory fitness (CRF) between the PAG and CON (Schrenk et al. 2025). However, exploratory within‐group analyses indicated that CRF values were higher at post‐intervention in the PAG, while there was no change in the CON (Schrenk et al. 2025). Given that the present analysis is based on a subset of 69 participants, we provide a separate report of CRF values in this study. Baseline and post‐intervention standardized V̇O_2max_ values (*z*‐scores; unitless), along with brain volume metrics, are summarized in Tables 2 and 3, respectively.

**TABLE 2:** Pre‐intervention measures of standardized V̇O_2max_ (*z*‐scores; unitless), total intracranial volume (TIV), gray matter volume, and white matter volume in the physical activity group (PAG) and the active control group (CON).

Measure	Physical activity group (*n* = 36)	Active control group (*n* = 33)	*p*
V̇O_2max_ (*z*‐score; unitless)	1.28 ± 1.15	0.96 ± 0.93	0.221
Total intracranial volume (cm^3^)	1480.7 ± 147.2	1467.9 ± 158	0.728
Total grey matter volume (cm^3^)	605.7 ± 55.2	595.8 ± 52.9	0.448
Total white matter volume (cm^3^)	495.7 ± 56.4	495.7 ± 60.8	0.997

*Note:* Summary statistics (mean ± SD) and between‐group *p*‐values are reported. No significant differences were found between groups at baseline (all *p* > 0.05). VO_2_max values are *z*‐standardized (unitless) to account for age‐ and sex‐related differences.

**TABLE 3:** Post‐intervention measures of standardized VO_2_max (*z*‐scores; unitless), total intracranial volume (TIV), gray matter volume, and white matter volume in the physical activity group (PAG) and the active control group (CON).

Measure	Physical activity group (*n* = 36)	Active control group (*n* = 33)	*p*
V̇O_2max_ (*z*‐score; unitless)	1.39 ± 1.13	0.91 ± 1.12	0.1007
Total intracranial volume (cm^3^)	1483.2 ± 18	1468.8 ± 159.3	0.698
Total grey matter volume (cm^3^)	605.100 ± 55.397	595.282 ± 54.930	0.462
Total white matter volume (cm^3^)	496.648 ± 58.425	497.039 ± 61.096	0.978

*Note:* Summary statistics (mean ± SD) and between‐group *p*‐values are reported. No significant differences were found between groups at post‐intervention (all *p* > 0.05). VO_2_max values are *z*‐standardized (unitless) to account for age‐ and sex‐related differences.

### 2.3 BrainAGE Analysis

**FIGURE 1:**
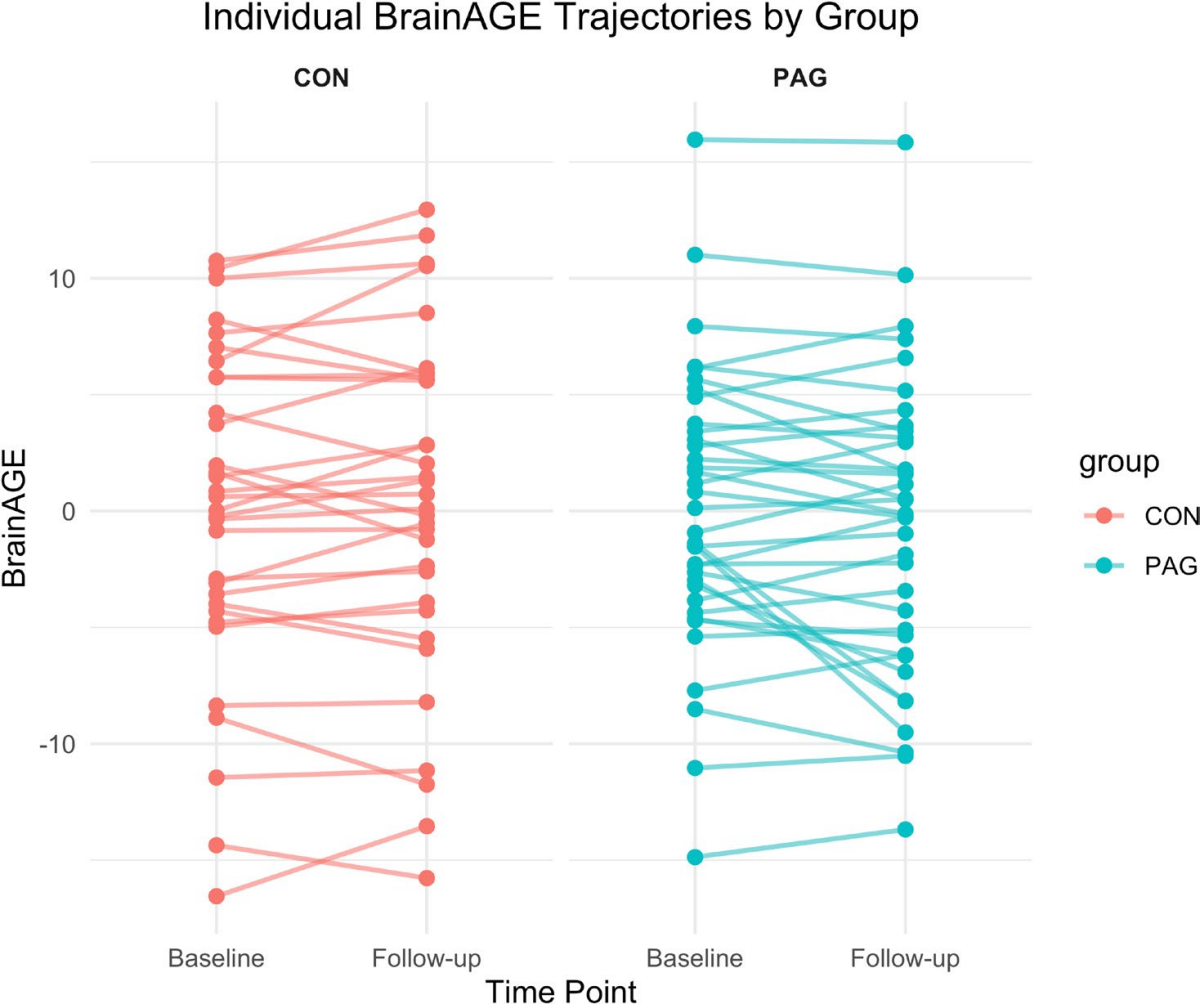
Individual changes in BrainAGE from baseline to follow‐up by group. Spaghetti plot showing each participant's BrainAGE at baseline and follow‐up, with lines connecting paired measurements to illustrate within‐person trajectories. Group means are overlaid as larger points. In the physical activity group (PAG), many participants show a downward trajectory (indicative of reduced BrainAGE), whereas changes in the active control group (CON) appear more variable and without a consistent pattern.

To visualize the initial and post‐intervention BrainAGE distribution, we generated box plots for baseline and follow‐up BrainAGE values across the entire cohort, separated by group (Figure 1). At baseline, both groups showed similar median BrainAGE values, indicating comparable starting points in terms of brain health relative to chronological age. At follow‐up, the physical activity group (PAG) showed a noticeable reduction in BrainAGE, while the control group (CON) displayed minimal change over time. The visualized distributions provide an overview of BrainAGE trends in each group throughout the study period.

The mean change in BrainAGE for the PAG group was −0.713 (SD = 6.42), while that in the CON was 0.295 (SD = 7.35). The baseline and follow‐up summary statistics, along with the calculated longitudinal changes, are presented in Table 4. These results suggest a more pronounced improvement in BrainAGE for the PAG group.

**TABLE 4:** Summary of BrainAGE at baseline, follow‐up, and longitudinal changes by group.

Group	Time point	Mean BrainAGE	SD	*N*
Physical activity group (PAG)	Baseline	0.005	6.048	36
	Follow up	−0.712	6.423	
	Change	−0.718	2.392	
Active control group (CON)	Baseline	−0.065	7.007	32
	Follow up	0.229	7.347	
	Change	0.294	1.792	

*Note:* This table presents the BrainAGE summary statistics at baseline, follow‐up, and the calculated longitudinal changes (follow‐up minus baseline) for both the training and control groups. The data provide insight into the differences in brain health changes between the groups over time.

To assess the impact of the intervention on BrainAGE over time, we conducted a series of regression analyses. The primary linear model included Group (PAG vs. CON), Sex, V̇O_2max_ Change, and baseline standardized V̇O_2max_. This model revealed a statistically significant association between group and BrainAGE changes (*β* = 1.101, *p* = 0.041), suggesting that participants in the PAG group experienced greater reductions in BrainAGE compared to the CON. However, V̇O_2max_ change was not a significant predictor (*β* = 0.119, *p* = 0.284), indicating that other factors may have contributed to the observed group effect. To explore potential moderation effects, interaction terms were included in additional models (V̇O_2max_ change × group, V̇O_2max_ change × sex, group × sex, and a three‐way interaction). None of these interactions reached statistical significance, suggesting that the intervention's impact on BrainAGE was consistent across sexes and V̇O_2max_ change levels.

**FIGURE 2:**
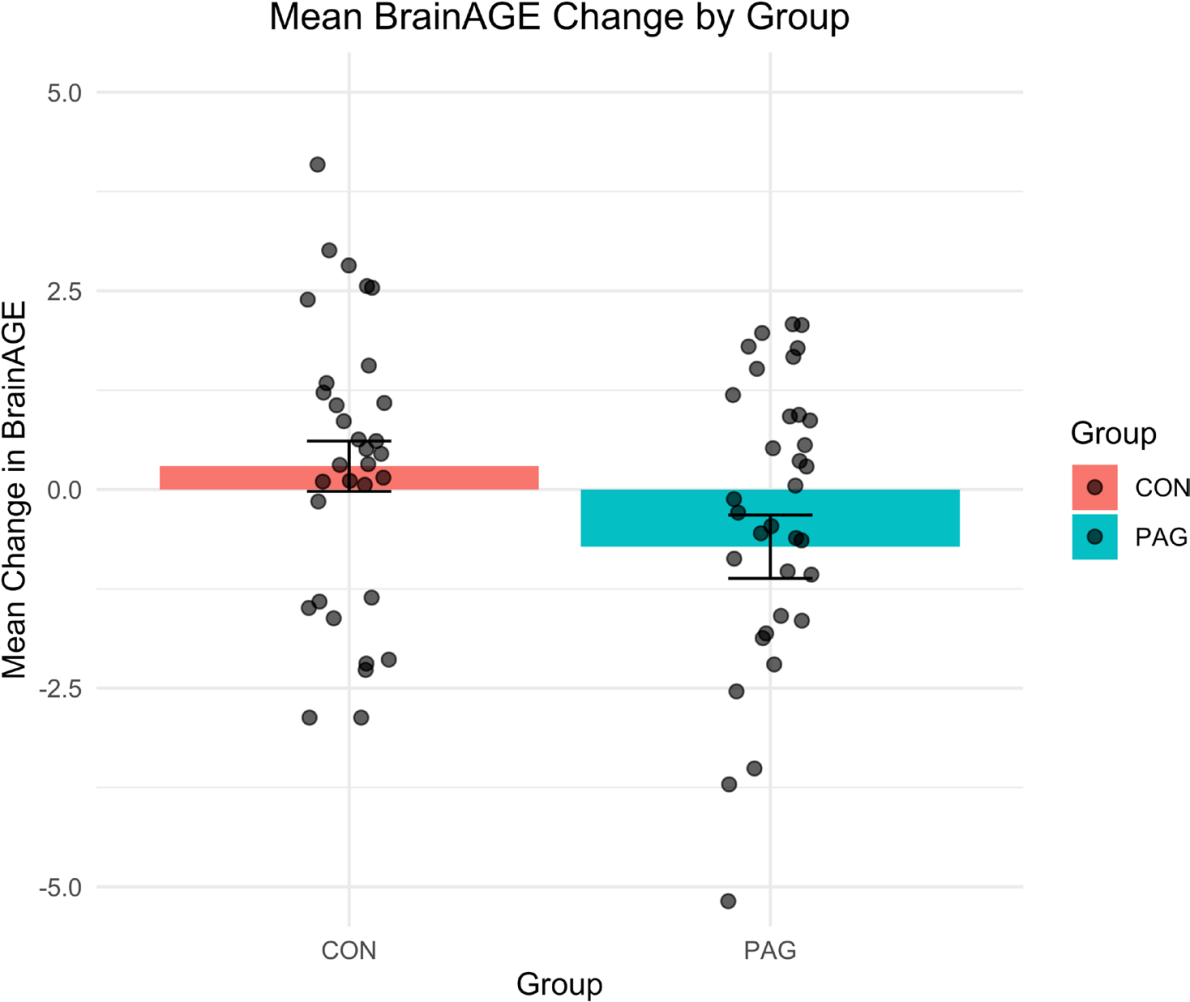
Change in BrainAGE from baseline to follow‐up by group. Boxplots depict the distribution of BrainAGE change scores (follow‐up minus baseline, in years) for the physical activity (PAG) and active control (CON) groups. Negative values indicate a reduction in BrainAGE. Boxes show the interquartile range (IQR), horizontal lines indicate medians, and whiskers represent 1.5 × IQR. Individual participants are shown as jittered points. The PAG group demonstrates a greater reduction in BrainAGE compared to the CON group.

Figure 2 further illustrates this finding with boxplots of the BrainAGE change, showing a reduction for the PAG and minimal change in the CON. Detailed statistical results are provided in Tables S1a–c. In addition, regression models stratified by baseline V̇O_2max_ quartiles within the PAG were conducted to align with VBM subgroup analyses. These models did not reveal statistically significant effects but are included in Tables S2a,b for completeness.

### 2.4 Voxel‐Based Morphometry (VBM) Findings

To investigate structural brain changes following the intervention, voxel‐based morphometry (VBM) analyses were conducted using CAT12. A full factorial design was first applied to gray matter (GM) difference images (follow‐up minus baseline), comparing PAG and CON. This analysis revealed no significant group‐level differences in GM volume change between PAG and CON. We next explored whether baseline fitness levels within the PAG modulated the effects of the intervention. Participants in the PAG were stratified into quartiles based on baseline V̇O_2max_, and a second VBM analysis was conducted using a full factorial design with these quartiles as the grouping factor. Significant results were found in the contrast comparing Q1 (lowest V̇O_2max_) with Q2 (next‐lowest V̇O_2max_), with GM volume increases in Q1 surviving threshold‐free cluster enhancement (TFCE) with family‐wise error (FWE) correction at *p* < 0.05. Effect size estimates indicated moderate differences between Q1 and Q2 for the significant clusters (Cluster 1: *g* = 0.60 [−0.38, 1.57]; Cluster 2: *g* = 0.75 [−0.23, 1.74]), although the wide confidence intervals reflect the small sample size (*n* = 9 per group).

**FIGURE 3:**
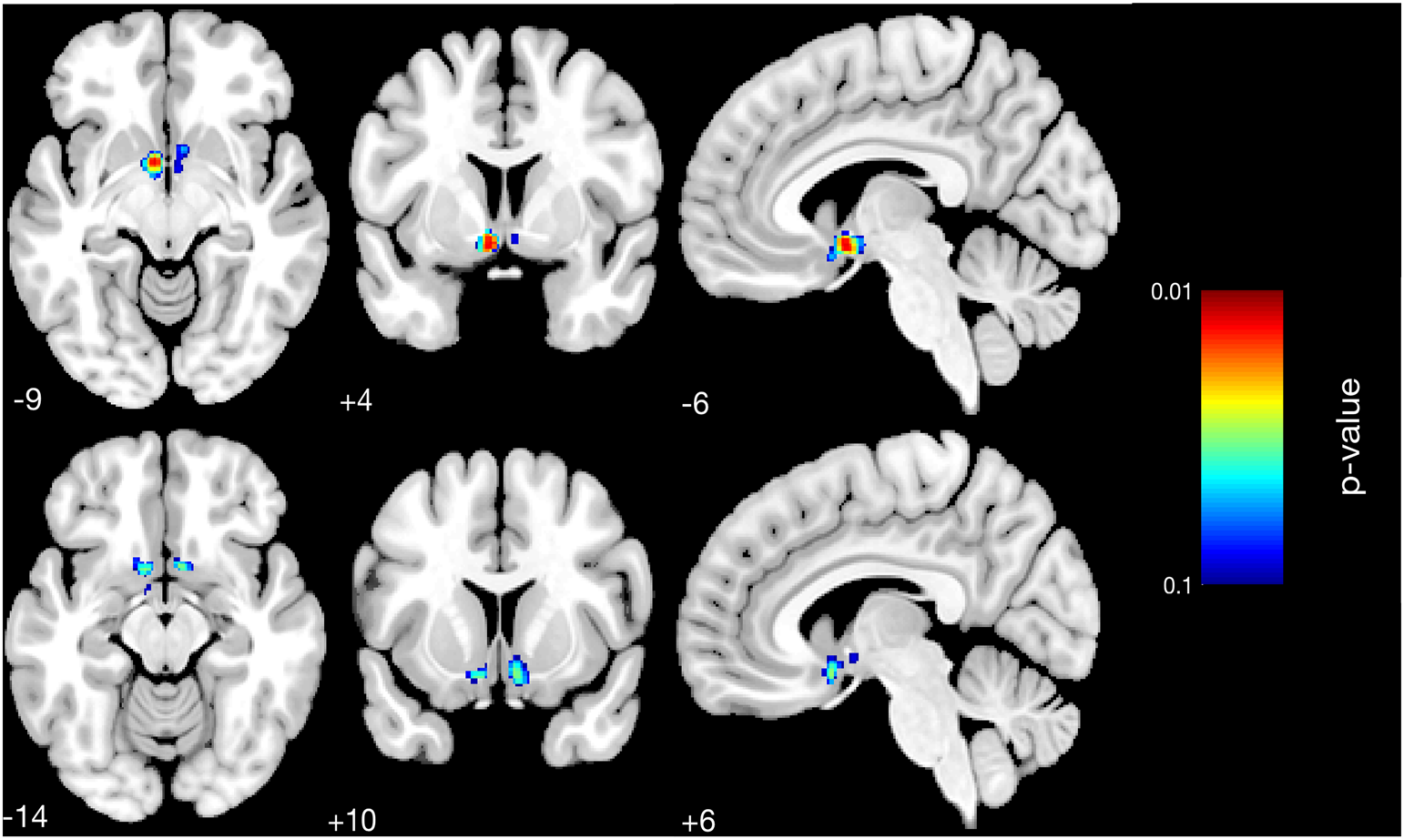
Results of voxel‐based morphometry (VBM) analysis for the physical activity group (PAG), showing brain regions with structural changes between Q1 and Q2 participants based on pre‐intervention V̇O_2_max. Significant clusters (*p* < 0.05, TFCE FWE‐corrected) are overlaid on a standard brain template. Highlighted areas include the left accumbens, left ventral diencephalon, left basal forebrain, right subcallosal area, and right accumbens. Corresponding effect sizes for these clusters are provided in Table 5.

These findings suggest that participants with lower baseline fitness levels exhibited greater neuroanatomical plasticity in response to the intervention. Significant clusters were located in the left accumbens area, left ventral diencephalon (DC), left basal forebrain, right subcallosal area, and right accumbens area (Table 5, Figure 3). No significant changes were observed in regions traditionally associated with aging, such as the hippocampus. Additional subgroup analyses, including a female‐only analysis, did not yield significant effects.

**TABLE 5:** Significant results of voxel‐based morphometry (VBM) analysis for the physical activity group (PAG).

*p*	Cluster size	*xyz* (mm)	Overlap of atlas region		Hedges' *g* (95% CI)
0.013	135	−6 4 9	7%	Left accumbens area	0.60 (−0.38, 1.57)
			7%	Left ventral diencephalon	
			6%	Left basal forebrain	
0.031	28	6 10 14	25%	Right subcallosal area	0.75 (−0.23, 1.74)
			14%	Right accumbens area	

*Note:* This table presents the significant clusters from the VBM analysis comparing quartile 1 (Q1) and quartile 2 (Q2) of baseline V̇O_2_max within the physical activity group. Results survived TFCE FWE correction at *p* < 0.05. Clusters were located in the left accumbens area, left ventral diencephalon, left basal forebrain, right subcallosal area, and right accumbens area. For each cluster, Hedges' g (95% CI) is reported as a measure of effect size: Cluster 1: *G* = 0.60 [−0.38, 1.57]; Cluster 2: *G* = 0.75 [−0.23, 1.74].

## 3 Discussion

This study investigated the impact of an eight‐week, multi‐component physical activity intervention on BrainAGE and structural brain changes in older adults. Our findings highlight the potential of regular physical exercise to mitigate age‐related brain changes, with notable reductions in BrainAGE observed in the physical activity group (PAG) compared to the active control group (CON). As these effects were not found to be related to changes in cardiorespiratory fitness (CRF), additional mechanisms seem to contribute to the intervention's benefits.

### 3.1 BrainAGE as a Sensitive Biomarker for Brain Health

The significant reduction in BrainAGE among participants of the PAG group suggests that physical activity may have a beneficial effect on brain health, reflected in a slowing of brain aging. This finding aligns with previous research linking physical activity to improved brain structure and function. BrainAge, as a composite measure of brain structure derived from advanced machine learning algorithms (Bittner et al. 2021; Franke et al. 2013; Ouyang et al. 2024), appears sensitive enough to detect these subtle neurobiological changes. Our results support the potential of physical activity to influence brain aging and reinforce the value of it as an outcome measure for evaluating intervention effects (Angoff et al. 2022; Phillips 2017; Shen et al. 2023; Voss et al. 2013).

### 3.2 Structural Brain Changes and Baseline CRF

Voxel‐based morphometry (VBM) analyses did not reveal significant group‐level differences in gray matter volume change between the physical activity group and the active control group. This null finding may reflect the relatively short duration of the intervention or ceiling effects due to the already high baseline fitness levels in our cohort (Langlois et al. 2013). While aging‐related brain regions such as the hippocampus have been frequently studied in this context, no significant structural changes were observed in those areas.

To explore whether baseline fitness levels modulate structural brain changes, we conducted additional analyses within the physical activity group based on pre‐intervention V̇O_2max_. Significant differences in gray matter volume change were observed only in the contrast between the two lowest fitness quartiles. Clusters were located in regions including the accumbens area, ventral diencephalon, and basal forebrain.

While these regions have been associated with affective and reward‐related processing—particularly motivation, regulation, and reinforcement learning (Haber and Knutson 2010; Rolls 2015)—further research is needed to clarify their precise role in physical activity‐induced neuroplasticity. These findings tentatively suggest that baseline cardiorespiratory fitness may influence responsiveness to exercise‐based interventions. However, given the absence of group‐level differences between PAG and CON, such subgroup effects should be interpreted cautiously (Franklin et al. 2023; Guiney and Machado 2013; Langlois et al. 2013).

### 3.3 Limitations and Future Directions

While our findings underscore the benefits of physical activity for brain health, several limitations should be acknowledged. The relatively short duration of the intervention and the high baseline fitness levels of our cohort may have constrained the scope for detecting more pronounced effects. Additionally, our small sample size and homogeneity in baseline characteristics limit the generalizability of the findings. Future studies should recruit larger, more diverse cohorts and extend intervention durations to capture broader and longer‐term benefits. Investigating additional biomarkers and mediators will also be critical for understanding the multifaceted effects of physical activity on brain health.

## 4 Materials and Methods

### 4.1 Participants and Study Design

**FIGURE 4:**
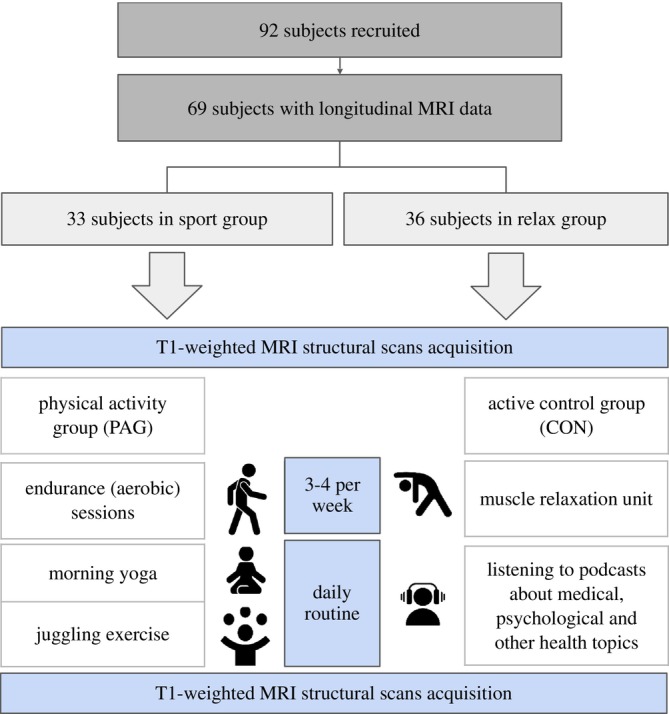
The first group's plan includes two main parts: (i) daily routines from monday to friday with morning yoga (5–10 min) and juggling exercise (5 min) starting with one ball up to three balls depending on the progress over 8 weeks and (ii) three times a week endurance (aerobic) sessions (30–40 min) with brisk walking or hiking. The second group received a plan consisting of (i) listening to podcasts about medical, psychological, and other health topics (10–15 min) and (ii) muscle relaxation unit (30–40 min) including sitting or lying down.

This study utilized T1‐weighted MR images from a monocentric, single‐blinded, randomized, actively controlled intervention study conducted by Jena University Hospital/Friedrich‐Schiller‐University (Schrenk et al. 2023). The primary objective was to investigate the impact of physical activity on brain health and cognition in older adults. A total of 92 healthy participants aged 60–75 years were recruited, with exclusion criteria including psychiatric or neurological conditions, cognitive impairment, and excessive alcohol or drug consumption (Figure 4). Detailed exclusion criteria are provided in Table S4. Baseline assessments included neurocognitive tests, structural and functional brain imaging, cardiorespiratory fitness tests, and gut‐microbiome sampling. Anthropometric measures (e.g., body mass, height, and waist circumference), submaximal exercise heart rate, and heart rate variability under resting conditions were also recorded. To minimize cardiovascular risks, participants underwent a medical checkup before starting the intervention. Participants were randomized into either a multicomponent training group or an active control group. Both groups followed an eight‐week intervention plan with equal time commitments. All intervention components and assessments were conducted entirely online. Post‐intervention, all assessments were repeated.

The physical activity group (PAG) engaged in aerobic exercises (medium‐ and high‐intensity walking), balancing exercises (yoga), and coordination exercises (juggling), aiming to improve cardiorespiratory fitness (V̇O_2max_ and submaximal exercise heart rate). The active control group (CON) performed progressive muscle relaxation exercises and listened to podcasts on aging, designed to equalize the time commitment without targeting fitness improvements.

This analysis focused on MRI‐derived BrainAGE changes and structural brain changes. A comprehensive description of all outcome measures is available in the trial protocol paper (Schrenk et al. 2023).

### 4.2 Assessment of Cardiorespiratory Fitness

As outlined in Schrenk et al. (2023), Cardiorespiratory fitness (CRF) was assessed by predicting maximum oxygen uptake (V̇O_2max_) via the submaximal Ekblom Bak cycle ergometer test, using an electronically braked cycle‐ergometer (ergoselect 100, Ergoline, Bitz, Germany) in a controlled laboratory setting (Björkman et al. 2016; Borg 1970; Ekblom‐Bak et al. 2014). During the 8‐min test, participants maintained a constant cadence of 60–65 RPM. The initial 4 min were at a fixed low power output of 30 watts, followed by 4 min at a higher, individually tailored power output targeting a perceived exertion level of approximately 14 on the Borg 6–20 scale (Borg 1982). Ratings of perceived exertion (RPE) were recorded in the second, third, and fourth minutes of each exercise stage. Heart rate was continuously monitored, and capillary lactate blood samples were collected at rest, in the final minute of each exercise stage, and at 1 and 3 min after cessation of the test. V̇O_2max_ was predicted using sex‐specific equations incorporating age, exercise heart rate difference between the predefined low standard power output (30 W), and a higher individually chosen power output (Björkman et al. 2016). The heart rate from the last 2 min of the second stage of the exercise test was defined as the “submaximal exercise heart rate” and thus used as a second index for the measurement of cardiorespiratory fitness. Additionally, CRF was estimated using a 6‐min walking test, during which participants covered as much distance as possible along a specified 20‐m route. Submaximal exercise heart rate and distance were continuously monitored, with post‐test assessments, including blood samples and subjective exhaustion scores, conducted 1 and 3 min after the trial (Schrenk et al. 2023). At last, a non‐exercise‐based prediction model was used, specifying sports habits, anthropometric measures, and resting sitting heart rate to estimate a person's V̇O_peak_, which is expressed in mL kg^−1^ min^−1^, that is, adjusted for body weight. For all statistical analyses and descriptive summaries, V̇O_2max_ values were *z*‐standardized (unitless) to account for age‐ and sex‐related differences.

### 4.3 MRI Scans Acquisitions and Preprocessing

For this study, all examinations were performed on a 3.0‐T MR scanner (Trio, Siemens, Erlangen, Germany) to obtain echo‐planar T2*‐weighted image volumes (EPI) and transaxial T1‐weighted structural images. T1‐weighted anatomical 3D images were collected following scan parameters: TR = 2.25 s, TE = 3.03 ms, inversion time, TI = 900 ms, field of view, FoV = 256 × 256 mm^2^, flip angle = 9°, voxel resolution = 1 × 1 × 1 mm^3^, and 176 axial slices.

All neuroimaging MR data were processed and analyzed with the Computational Anatomy Toolbox (CAT12.8.2 (Gaser et al. 2024); https://www.neuro.uni‐jena.de/cat) and Statistical Parametric Mapping (SPM12 (Welcome Center for Human Neuroimaging, n.d.; https://www.fil.ion.ucl.ac.uk/spm/); Wellcome Department of Cognitive Neurology) running under MATLAB 18a. CAT12's longitudinal preprocessing pipelines optimized for detecting small volumetric changes (e.g., brain plasticity, neuroplasticity workflow) were used with default settings. A detailed description of the individual preprocessing steps can be found in the CAT12 Manual (Gaser et al. 2024).

For each participant, baseline and follow‐up images were co‐registered and then realigned across the entire sample. The preprocessing workflow encompassed bias correction, image segmentation into cerebrospinal fluid, white matter, and gray matter, and transformation into MNI space using Shooting registration. Delta images (i.e., “follow‐up” minus “baseline”) were generated using CAT12's cat_stat_diff function. These images were smoothed with a 6‐mm Gaussian FWHM kernel to prepare for subsequent statistical analyses. Given the focus of our analysis, we did not use the baseline images directly but instead focused on the delta images. The use of delta images is advantageous as it allows for a more straightforward regression analysis with other parameters while maintaining the longitudinal nature of the study. By analyzing the changes between time points rather than static images, we can more effectively assess the impact of the intervention on brain structure over time.

### 4.4 BrainAGE Estimation

In this study, we adopted the newly updated BrainAGE framework, as recently outlined in the literature, which promises enhanced accuracy over its predecessors (Kalc et al. 2024). This advanced framework utilizes Gaussian process regression (GPR) optimized via the conjugate gradient method, coupled with an ensemble of models to reduce overfitting and refine predictive performance. For preprocessing, dimensionality reduction techniques including smoothing, downsampling, and principal component analysis (PCA) were implemented. The model ensemble was trained on affine‐registered segments of gray matter (GM) and white matter (WM), each model varying in preprocessing parameters to fine‐tune accuracy. The GPR was configured with a linear covariance function, a constant mean function set at 100, and a Gaussian likelihood function, with the hyperparameter adjusted to −1 (Rasmussen 2004). Adjustments for age bias in the BrainAGE estimates were made using a linear term, following the methodology proposed by Smith et al. (2019).

### 4.5 Statistical Analysis

Statistical analyses were conducted using R (version 3.6.2) (Rdc 2010) for descriptive statistics and linear regression models, alongside CAT12 under MATLAB VBM. Initial descriptive statistics provided insights into baseline characteristics of participants, including the distribution of BrainAGE, establishing a foundational understanding of the cohort's initial conditions. Additional data supporting the statistical analyses, including complete regression outputs and group‐level descriptive statistics, are available in Tables S1a–e.

### 4.6 Voxel‐Based Morphometry (VBM)

Voxel‐based morphometry (VBM) analyses were conducted using CAT12 on difference images (baseline and follow‐up) for each participant. An initial analysis employed a full factorial design with one factor (Group) consisting of two levels: PAG and CON. The aim was to assess overall differences in gray matter (GM) volume changes between groups.

After this initial whole‐group comparison yielded no significant differences, we further stratified within the PAG based on baseline V̇O_2max_ values. Using R's ntile function, the sport group was divided into quartiles (*n* = 4) according to pre‐intervention V̇O_2max_ levels. A second VBM analysis with a full factorial design was conducted exclusively within the PAG, hypothesizing that baseline fitness levels may modulate neuroplasticity. The factor for this design included four levels (quartiles of V̇O_2max_) with equal variance and independence specified, per CAT12's guidelines for full factorial ANOVA. Covariates (e.g., age, sex) were included as specified in the analysis.

### 4.7 Regression Analysis

The regression analyses included 68 participants due to one participant's missing data on BrainAGE. V̇O_2max_ values used in all analyses were *z*‐standardized (unitless) to account for age‐ and sex‐related differences. Additional statistical adjustment for body mass in the regression models did not materially change the results; therefore, the simpler model was retained for clarity. Linear regression models were used to explore the effects of the physical activity intervention on BrainAGE changes. The primary model included Group (PAG vs. CON), sex, V̇O_2max_ change, and baseline standardized V̇O_2max_ as predictors. Interaction terms, such as V̇O_2max_ change * group, group * sex, and V̇O_2max_ change * sex, were incorporated into subsequent models to examine potential moderation effects. These interaction models allowed for an in‐depth analysis of whether the intervention's impact on BrainAGE varied across different subgroups. Baseline V̇O_2max_ was included as a covariate in the primary model to control for initial fitness levels. In the interaction models, V̇O_2max_ Change was used to assess differential effects of the intervention on BrainAGE progression across subgroups. To align with the VBM findings, an additional model stratified the training group into quartiles based on baseline V̇O_2max_, investigating the differential effects of baseline fitness levels on BrainAGE changes. Quartile‐specific models were also run to explore within‐group variability.

Box plots were generated to visually depict BrainAGE distributions and changes, both by group (PAG vs. CON) and by baseline fitness quartiles (Figures 1, 2, 3). These visualizations provided additional context, illustrating group differences and trends consistent with the regression findings. While the primary regression model showed a significant group effect on BrainAGE change, exploratory models stratified by baseline V̇O_2max_ quartiles did not yield significant results. These are provided in Tables S2a,b for transparency and to complement the VBM findings, which also focused on pre‐intervention fitness levels as a potential moderator of training effects.

## Supporting information


**Data S1:** Supporting Information.

## Data Availability

The data that support the findings of this study were obtained from a third party and are not publicly available. Access to these data may be granted upon reasonable request and with permission from Simon Schrenk (simon.schrenk@med.uni‐jena.de). The data were used with permission for this study.
