# Supplementary material for: Physical Activity and BrainAGE: Exploring the Impact on Brain Health and Plasticity in Older Adults
Source: Hum Brain Mapp. 2025 Oct 14;46(15):e70378. doi: 10.1002/hbm.70378 (PMC12519997; doi:10.1002/hbm.70378)
Supplement: Supplementary file 1 — Data S1: Supporting Information. [file HBM-46-e70378-s001.docx]

# **Supplementary**

**Supplementary Table S1a: Primary Model**

| **Predictor** | **Estimate (β)** | **Std. Error** | **t-value** | **p-value** |
| --- | --- | --- | --- | --- |
| **(Intercept)** | -2.27013 | 0.99025 | -2.292 | 0.0252 * |
| **V̇O_2max_ Change (Main Effect)** | 0.11931 | 0.11034 | 1.081 | 0.2837 |
| **Group (PAG vs CON)** | 1.1012 | 0.5289 | 2.082 | 0.0414 * |
| **Sex (Male vs Female)** | 1.0466 | 0.6145 | 1.703 | 0.0935 . |
| **V̇O_2max_ Pre-intervention** | 0.04102 | 0.26404 | 0.155 | 0.8770 |

**Description**: This table presents the primary regression model, showing Group as a significant predictor of BrainAGE change, indicating the intervention’s direct effect. Pre-intervention V̇O_2max_ included as a covariate. PAG: physical activity group; CON: active control group

#### **Supplementary Table S1b: Interaction Models**

1. **Model 1 - V̇O_2max_ Change * Sex Interaction**

| **Predictor** | **Estimate (β)** | **Std. Error** | **t-value** | **p-value** |
| --- | --- | --- | --- | --- |
| **(Intercept)** | -2.1447 | 0.8243 | -2.602 | 0.0115 * |
| **V̇O_2max_ Change (Main Effect)** | 0.2879 | 0.1607 | 1.791 | 0.0781 . |
| **Sex (Male vs Female)** | 0.9759 | 0.5551 | 1.758 | 0.0836 . |
| **V̇O_2max_ Change * Sex (Interaction)** | -0.3099 | 0.2176 | -1.424 | 0.1594 |

**Description:** This table displays the results of a linear regression model assessing the interaction between V̇O_2max_ Change and Sex, alongside their main effects and Group as a covariate. This model evaluates whether the relationship between V̇O_2max_ Change and BrainAGE differs by Sex, providing insights into potential gender-specific responses to the intervention.

1. **Model 2 - V̇O_2max_ Change * Group Interaction**

| **Predictor** | **Estimate (β)** | **Std. Error** | **t-value** | **p-value** |
| --- | --- | --- | --- | --- |
| **(Intercept)** | -2.1773 | 0.8392 | -2.595 | 0.0118 * |
| **V̇O_2max_ Change (Main Effect)** | 0.0587 | 0.3835 | 0.153 | 0.8788 |
| **Group (PAG vs CON)** | 1.0826 | 0.5220 | 2.074 | 0.0422 * |
| **V̇O_2max_ Change * Group (Interaction)** | 0.0369 | 0.2244 | 0.165 | 0.8698 |

**Description:** This table reports the linear regression model incorporating the interaction term between V̇O_2max_ Change and Group, along with their main effects and Sex as a covariate. The interaction term tests whether the relationship between V̇O_2max_ Change and BrainAGE is modified by the intervention group (training vs. control), helping to identify if V̇O_2max_ changes have a differential impact on BrainAGE across groups. PAG: physical activity group; CON: active control group

**Supplementary Table S1c: Extended Interaction Models**

1. **Model 3 - Group * Sex Interaction**

| **Predictor** | **Estimate (β)** | **Std. Error** | **t-value** | **p-value** |
| --- | --- | --- | --- | --- |
| **(Intercept)** | -1.9688 | 0.9832 | -2.002 | 0.0495 * |
| **V̇O_2max_ Change (Main Effect)** | 0.1283 | 0.1123 | 1.143 | 0.2575 |
| **Group (PAG vs CON)** | 0.9349 | 0.6333 | 1.476 | 0.1449 |
| **Sex (Male vs Female)** | 0.3294 | 1.7014 | 0.194 | 0.8471 |
| **Group * Sex (Interaction)** | 0.4688 | 1.1085 | 0.423 | 0.6738 |

**Description:** This table provides results for the regression model evaluating the interaction between Group and Sex, alongside their main effects and V̇O_2max_ Change as a covariate. The interaction term examines whether the effect of the intervention (Group) on BrainAGE changes varies between males and females, shedding light on potential sex-specific intervention effects.PAG: physical activity group; CON: active control group

1. **Model 4 - V̇O_2max_ Change * Group * Sex Interaction**

| **Predictor** | **Estimate (β)** | **Std. Error** | **t-value** | **p-value** |
| --- | --- | --- | --- | --- |
| **(Intercept)** | -2.09207 | 1.02249 | -2.046 | 0.0451 * |
| **V̇O_2max_ Change (Main Effect)** | 0.34697 | 0.52925 | 0.656 | 0.5146 |
| **Group (PAG vs CON)** | 0.96319 | 0.65687 | 1.466 | 0.1478 |
| **Sex (Male vs Female)** | 0.62403 | 1.75802 | 0.355 | 0.7239 |
| **V̇O_2max_ Change * Group (Interaction)** | -0.03867 | 0.32823 | -0.118 | 0.9066 |
| **V̇O_2max_ Change * Sex (Interaction)** | -0.82370 | 0.78962 | -1.043 | 0.3011 |
| **Group * Sex (Interaction)** | 0.3012 | 1.1586 | 0.260 | 0.7958 |
| **V̇O_2max_ Change * Group * Sex (3-way interaction)** | 0.3123 | 0.46904 | 0.666 | 0.5081 |

**Description**: This table summarizes the regression model with a three-way interaction term (V̇O_2max_ Change * Group * Sex), in addition to the main effects and two-way interaction terms. The three-way interaction assesses whether the combined effects of V̇O_2max_ Change and Group on BrainAGE differ by Sex, offering insights into complex subgroup-specific relationships influenced by the intervention. PAG: physical activity group; CON: active control group

**Supplementary Table S2a: Subgroup Regression Models by Quartile (Physical Activity Group)**

| **Quartile (Q)** | **Predictor** | **Estimate (β)** | **Std. Error** | **t-value** | **p-value** |
| --- | --- | --- | --- | --- | --- |
| **Q1** | **(Intercept)** | -0.9500 | 0.7017 | -1.354 | 0.218 |
|  | **Sex (Male vs Female)** | -0.1240 | 0.9414 | -0.132 | 0.899 |
| **Q2** | **(Intercept)** | 0.2325 | 0.8434 | 0.276 | 0.791 |
|  | **Sex (Male vs Female)** | 0.3975 | 1.1316 | 0.351 | 0.736 |
| **Q3** | **(Intercept)** | -2.322 | 1.573 | -1.476 | 0.190 |
|  | **Sex (Male vs Female)** | 2.092 | 3.146 | 0.665 | 0.531 |
| **Q4** | **(Intercept)** | -1.2475 | 0.7688 | -1.623 | 0.149 |
|  | **Sex (Male vs Female)** | NA | NA | NA | NA |

**Description:** This table presents regression results for BrainAGE changes within each quartile of pre-intervention VO₂max for the physical activitygroup. Coefficients, standard errors, t-values, and p-values for the intercept and sex are included for each quartile, offering insights into potential subgroup-specific effects..

**Supplementary Table S2b: Supplementary Table S1d: Regression Model for BrainAGE Change in Quartiles (Physical Activity Group)**

| **Quartile (Q)** | **Predictor** | **Estimate (β)** | **Std. Error** | **t-value** | **p-value** |
| --- | --- | --- | --- | --- | --- |
| **Reference (Q1)** | **(Intercept)** | -1.3689 | 0.9498 | -1.441 | 0.160 |
| **Q2** | **V̇O_2max_ Pre-intervention** | 1.4722 | 1.1089 | 1.328 | 0.195 |
| **Q3** | **V̇O_2max_ Pre-intervention** | -0.5873 | 1.1804 | -0.498 | 0.623 |
| **Q4** | **V̇O_2max_ Pre-intervention** | 0.1214 | 1.2624 | 0.096 | 0.924 |
| **All Quartiles** | **Sex (Male vs Female)** | 0.6300 | 0.9648 | 0.653 | 0.519 |

**Description:** This table summarizes the results of the linear regression model for BrainAGE changes across quartiles of pre-intervention VO₂max within the training group. The table provides coefficient estimates, standard errors, t-values, and p-values for each predictor, illustrating the relationship between BrainAGE changes and baseline fitness levels.

**Supplementary Table S3. Regular Physical Activity Prescription**

| **Component** | **Type** | **Frequency** | **Duration** | **Description** |
| --- | --- | --- | --- | --- |
| **Aerobic Exercises** | Medium-Intensity Walking | 3 times/week | 30 min | Borg Scale: 2–3 |
| **Aerobic Exercises** | High-Intensity Walking | 2 times/week | 20 min | Borg Scale: 4 |
| **Balancing Exercises** | Yoga | 2 times/week | 40 min | Focus on balance and flexibility |
| **Coordination** | Juggling | 3 times/week | 10 min | Skill-building activity |

**Supplementary Table S4. Exclusion criteria for participants**

| 1. Relevant, uncorrected vision disorder |  |
| --- | --- |
| 2. Evidence of cognitive impairment (ACE-III score of ≤ 86) |  |
| 3. Relevant psychiatric or neurological pre-existing condition (e.g., major depression, focal damage of the brain due to operation or stroke, epilepsy) |  |
| 4. Contraindications for MRI (e.g., cardiac pacemaker, non-removable metal parts in/at the body, claustrophobia; excluded from gut-microbiome analyses only; included in remaining analyses) |  |
| 5. Permanent medication with sedating substances |  |
| 6. Loss of capacity of consent |  |
| 7. Relevant, physical impairment |  |
| 8. Stadium 3 and 4 cardiac diseases |  |
| 9. Relevant systemic disease: cancer, severe kidney or liver disease, type 1 or 2 diabetes, diseases with intrinsic inflammation |  |
| 10. Treatments that influence glucose or microbiome composition: antidiabetics, antibiotics, immune suppressants, etc. (excluded from gut-microbiome analyses only; included in remaining analyses) |  |
| 11. Chronic obstipation or diarrhea |  |
| 12. Symptoms of clinical infection during the last month |  |
| 13. Excessive alcohol/drug consumption |  |
| 14. Severe eating disorder |  |
| 15. Known disruption of iron balance |  |
| 16. Chronic treatment with steroidal anti-inflammatory drugs |  |
| 17. Treatment with proton pump inhibitors |  |
